# Significant Strain Variation in the Mutation Spectra of Inbred Laboratory Mice

**DOI:** 10.1093/molbev/msz026

**Published:** 2019-02-11

**Authors:** Beth L Dumont

**Affiliations:** The Jackson Laboratory, Bar Harbor, ME

**Keywords:** house mice, germline mutation, mutation spectrum

## Abstract

Mutation provides the ultimate source of all new alleles in populations, including variants that cause disease and fuel adaptation. Recent whole genome sequencing studies have uncovered variation in the mutation rate among individuals and differences in the relative frequency of specific nucleotide changes (the mutation spectrum) between populations. Although parental age is a major driver of differences in overall mutation rate among individuals, the causes of variation in the mutation spectrum remain less well understood. Here, I use high-quality whole genome sequences from 29 inbred laboratory mouse strains to explore the root causes of strain variation in the mutation spectrum. My analysis leverages the unique, mosaic patterns of genetic relatedness among inbred mouse strains to identify strain private variants residing on haplotypes shared between multiple strains due to their recent descent from a common ancestor. I show that these strain-private alleles are strongly enriched for recent de novo mutations and lack signals of widespread purifying selection, suggesting their faithful recapitulation of the spontaneous mutation landscape in single strains. The spectrum of strain-private variants varies significantly among inbred mouse strains reared under standardized laboratory conditions. This variation is not solely explained by strain differences in age at reproduction, raising the possibility that segregating genetic differences affect the constellation of new mutations that arise in a given strain. Collectively, these findings imply the action of remarkably precise nucleotide-specific genetic mechanisms for tuning the de novo mutation landscape in mammals and underscore the genetic complexity of mutation rate control.

## Introduction

The de novo mutation rate determines the frequency at which new alleles arise in populations, with the potential for such variants to drive adaptive evolution or cause disease. Knowledge of this fundamental quantity is critical for interpreting levels of neutral diversity in populations (Kimura 1983), dating historical events from genetic data (Scally and Durbin 2012; Moorjani et al. 2016), and forecasting the ultimate fate of a species (Lynch 2016). De novo mutation rates also determine the incidence of many rare Mendelian diseases (Kondrashov 2003) and influence genetic risk for multiple common diseases (Vissers et al. 2010; Girard et al. 2011; O’Roak et al. 2011, 2012; Xu et al. 2011; Iossifov et al. 2012; Neale et al. 2012; Sanders et al. 2012; Bomba et al. 2017).

Despite its critical importance to human health and evolution, the rate of mutation exhibits considerable variability. Mutation rates fluctuate across genomes (Hodgkinson and Eyre-Walker 2011; Ségurel et al. 2014), conditional on aspects of the local chromatin environment (Schuster-Böckler and Lehner 2012; Chen et al. 2017; Carlson et al. 2018), recombination rate (Lercher and Hurst 2002; Hardison et al. 2003; Besenbacher et al. 2016), GC content (Hardison et al. 2003; Schaibley et al. 2013), DNA replication timing (Stamatoyannopoulos et al. 2009; Francioli et al. 2015), transcription (McVicker and Green 2010; Park et al. 2012; Chen et al. 2017), and flanking nucleotide context (Duncan and Miller 1980; Hwang and Green 2004; Carlson et al. 2018). The mutation rate varies by orders of magnitude between species (Uchimura et al. 2015; Smeds et al. 2016; Senra et al. 2018), presumably reflecting species differences in DNA repair mechanisms (Hart and Setlow 1974; MacRae et al. 2015), metabolism (Martin and Palumbi 1993), and life history (Nabholz et al. 2008). There are striking mutation rate differences among individuals, including a marked dimorphism between males and females and a pronounced age effect (Crow 2000; Conrad et al. 2011; Kong et al. 2012; O’Roak et al. 2012; Francioli et al. 2015; Besenbacher et al. 2016; Jonsson et al. 2017). Population genomic analyses even point to differences in the mutation spectrum—the relative fraction of de novo mutations that result in particular types of nucleotide changes—among individuals (Harris 2015; Assaf et al. 2017; Harris and Pritchard 2017; Mathieson and Reich 2017). Thus, a subset of individuals in a population disproportionately contributes to the pool of new mutations that arise each generation, and different individuals are more prone to transmitting particular types of single nucleotide mutations to their offspring.

While differences in parental age at reproduction may account for much of the observed variation in human germline mutation rates (Kong et al. 2012; Jonsson et al. 2017), environmental and genetic factors likely also play a role. Exposure to ionizing radiation (Dubrova, Bersimbaev, et al. 2002; Dubrova, Grant, et al. 2002), cigarette smoke (Zenzes 2000; Shi et al. 2001; DeMarini 2004), caffeine (Robbins, Vine, et al. 1997), and chemotherapeutic agents (Robbins, Meistrich, et al. 1997; Frias et al. 2003) have been previously associated with increased germline mutation loads in humans. Differences in exposures among individuals could contribute to observed mutation rate variation. In addition, there are >1,000 genes in the mammalian genome with annotated functions in DNA damage surveillance, DNA repair, and the metabolism of genotoxic compounds (Carbon et al. 2009). These loci represent potential reservoirs of functional genetic variation modifying the fidelity and efficiency of DNA damage repair and tuning genomic sensitivity to mutagens (Baer et al. 2007). Consistent with this possibility, mutations in DNA repair genes have been associated with elevated *somatic* mutation rates (Ahn et al. 2016), increased cancer risk (Easton et al. 2007; Dowty et al. 2013), and premature aging (de Boer et al. 2002; Lombard et al. 2005). Despite the considerable functional overlap of the DNA proofreading and repair machinery between the soma and the germline, there have been few efforts to directly link variation at putative modifier loci to variation in germline mutation rates in mammals (Uchimura et al. 2015; Seoighe and Scally 2017).

Comparative genomic analyses of cancer tumors and control tissues have uncovered remarkably precise, sequence-dependent mechanisms of mutation. Defects in specific DNA repair genes and pathways can result in distinct somatic mutation signatures, defined by the relative enrichment of mutation events in specific nucleotide contexts (Nik-Zainal et al. 2012; Alexandrov et al. 2013; Helleday et al. 2014). For example, altered activity of the error prone polymerase Polε is associated with 5′-T**C**T-3′>5′-T**A**T-3′ and 5′-T**T**T-3′>5′-T**G**T-3′ mutations in human cancers (Cancer Genome Atlas Network 2012; Alexandrov et al. 2013; Cancer Genome Atlas Research Network 2013; Shinbrot et al. 2014). Cancers with altered AID/APOBEC mutational activity are characterized by a preponderance of 5′-T**C**A-3′>5′-T**T**A-3′ mutations (Nik-Zainal et al. 2012; Alexandrov et al. 2013). Such observations raise the possibility that segregating polymorphisms in DNA repair genes could also influence *germline* mutation rates with extraordinary sequence context precision. Indeed, there are significant differences in the human germline mutation spectrum inferred from population-private (Harris 2015), rare (Mathieson and Reich 2017), and derived alleles (Harris and Pritchard 2017). However, the challenge of disentangling variable parental age, differential environmental exposures, and genetic differences between human populations makes it difficult to address the underlying causes of observed variation in the mutation spectrum in our own species.

The availability of high-quality genomes from multiple inbred laboratory mouse strains provides a powerful opportunity to overcome this limitation (Keane et al. 2011; Adams et al. 2015). As a consequence of their unique historical origins from a small founder population (Wade and Daly 2005; Yang et al. 2007, 2011), variants that are private to single inbred mouse strains but that reside on haplotypes that are otherwise shared between strains have likely arisen de novo since their inception as laboratory models. I leverage this recognition to derive the germline mutation spectrum in 29 common inbred mouse strains. I document significant differences in the mutation spectrum between mouse strains reared in standard laboratory environments and show that this variation is not solely accounted for by differences in strain age at breeding. My findings suggest that multiple modifiers of the mutation spectrum segregate among inbred mouse strains, implying that the process of germline mutation is itself a complex genetic trait.

## Results and Discussion

### Identification of Recent, Spontaneous Mutations in Laboratory Mice

**Fig. 1.**
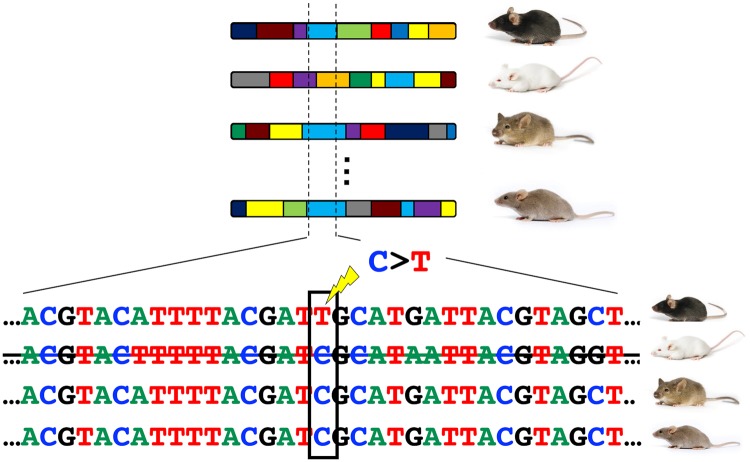
Schematic of the approach used to identify putative de novo mutations in inbred laboratory mouse strains. The genomes of laboratory mice can be envisaged as mosaics of less than ten haplotypes derived from a small population of founder animals. The ancestral haplotype structure of four strains is illustrated, with unique haplotypes depicted in different colors. Given the small number of founders, multiple strains are likely to share the same haplotype at a given locus by virtue of their descent from a common ancestor. An example of one such region shared by three of the four depicted strains (light blue haplotype) is outlined by dashed lines. Recent mutations that arose in a single focal strain can be detected as strain-private variants resident on haplotypes that are shared identical-by-descent between strains.

The classical laboratory mouse strains derive from a small, ancestral population of wild founder animals that were selectively bred for traits of interest by mouse fanciers in the late 19th and early 20th centuries. As a consequence of their unique historical origins, the genetic diversity captured in laboratory mice represents an extremely limited sample of the diversity found in wild mouse populations (Salcedo et al. 2007; Yang et al. 2011; Phifer-Rixey and Nachman 2015). Notably, variation among inbred strains at over 97% of the genome can be reconciled into fewer than ten distinct haplotypes (Yang et al. 2011). Thus, the genomes of the laboratory strains can be envisioned as mosaics derived from this small founder pool, with the haplotype of a given genomic region in one strain likely shared between multiple strains (fig. 1).

Although the majority of genetic variants segregating among the inbred laboratory strains trace to variation in this “Fancy Mouse” founder population, a small remaining number of sites can be attributed to recent de novo mutations that occurred following their inception as laboratory models. Many inbred strains carry known spontaneous mutations that drifted to fixation in inbreeding colonies. For example, a de novo mutation on the C57BL/6N background generated a missense mutation in *cytoplasmic FMRP interacting protein 2* (*Cflip2*) that confers a sensitized response to cocaine (Kumar et al. 2013). Similarly, a spontaneous mutation in *Tlr4* in C3H/HeJ mice renders this strain uniquely resistant to endotoxin (Qureshi et al. 1999).

One strategy for systematically identifying these recent, spontaneous mutations is to identify alleles that are private to a single laboratory mouse strain, but that reside on a haplotype that is otherwise identical by descent (IBD) in multiple strains (fig. 1). A conceptually similar approach has been previously used to identify de novo mutations in large, multigenerational human pedigrees (Campbell et al. 2012; Palamara et al. 2015; Narasimhan et al. 2017). To this end, I used publicly available high-quality whole-genome sequences from 29 inbred mouse strains to identify autosomal IBD regions ≥5 Mb shared between at least 2 strains (Keane et al. 2011; Adams et al. 2015; see Materials and Methods). On an average, 46.8% of the genome of a given inbred strain is present in IBD blocks of this size (range: 6.7% KK/HiJ to 82.7% C57BL/6NJ; supplementary table S3, Supplementary Material online). In contrast, no IBD blocks ≥5 Mb are observed in a sample of 27 wild *Mus musculus domesticus* genomes (supplementary table S2 and fig. S1, Supplementary Material online; Harr et al. 2016). These findings reinforce the close genetic relatedness of the common laboratory strains and confirm their origins from a very small number of founder individuals (Beck et al. 2000; Frazer et al. 2007; Yang et al. 2011).

I next identified 15,311 strain private variants (SPVs) on large (≥5 Mb) IBD blocks (fig. 1; see Materials and Methods). Approximately 91.7% of these SPVs are fixed for the alternative allele, with the remaining 8.3% present in a heterozygous state. On an average, there are 528 SPVs per strain (Range: 103 in KK/HlJ to 1,028 C57BR/cdJ; supplementary tables S3 and S4, Supplementary Material online), with the number of SPVs strongly correlated with the fraction of a given strain’s genome captured in IBD regions (Spearman’s Rho = 0.824; *P *=* *4.0×10^−8^). Given the absence of IBD blocks ≥5 Mb in wild mouse populations (supplementary table S2 and fig. S1, Supplementary Material online), these SPVs are almost certainly due to recent mutations, as opposed to variants inherited from wild mouse ancestral populations.

### Strain Private Variants Approximate Neutral Expectations

The maintenance of inbred strains via small “foundation stocks” of sister-brother mating pairs approximates the experimental design of a mutation accumulation experiment and minimizes the efficacy of natural selection at each generation (Currer et al. 2009). Consequently, the vast majority of new mutations that arise during strain propagation are expected to be effectively neutral and their ultimate fate will be governed by chance (Eyre-Walker and Keightley 2007). Only a small subset of these neutral mutations will drift to fixation within an inbreeding colony, but those that do fix should be a representative, random sample of all neutral mutations. In contrast, a small number of new mutations are expected to be deleterious, and even fewer will confer an adaptive advantage. Although nonneutral mutations will be subject to selection or go unrealized due to viability defects or infertility, such large-effect variants should comprise a small fraction of all new mutations (Eyre-Walker and Keightley 2007; Uchimura et al. 2015). Based on these considerations, I reasoned that the set of SPVs for a given strain should approximate the strain-specific distribution of germline mutations.

To confirm the interpretation of observed SPVs as recent de novo mutations that have not been strongly biased by natural selection, I took advantage of expected differences in signals of historical selection at young versus old mutations. Mutations that have arisen recently in the laboratory strains have not been segregating for ample time to bear strong signatures of past selection. As a result, new mutations should be approximately randomly distributed within functional genomic regions. In contrast, old ancestral variants have been subject to generations of purifying selection and should be depleted for functional variants. I tested these dueling predictions using two approaches.

First, I determined the fraction of coding SPVs that result in synonymous and nonsynonymous changes. Assuming a uniform probability of mutation at all amino acid encoding sites, 72.2% and 3.8% of spontaneous mutations should result in missense and nonsense changes, respectively (Assaf et al. 2017). In rough agreement with these null expectations, 67.2% and 3.4% of the coding SPVs identified on IBD haplotypes are missense and nonsense substitutions, respectively (table 1). This represents a significant enrichment for potentially functional protein-coding variation relative to common variants segregating in ≥2 mouse strains (*G*-test, *P *=* *8.22×10^−60^; table 1). The observed deficit of nonsynonymous substitutions relative to the expected 72.2% may be explained by selection against mutations that confer lethality, infertility, or alter stereotyped strain phenotypes. Importantly, these bulk trends are broadly recapitulated on a per strain basis: the frequencies of missense, nonsense, and synonymous SPVs within coding regions do not significantly differ from null expectations for 27 of the 29 inbred strains (*G*-test of independence, *P *>* *0.05; supplementary table S5 and fig. S2, Supplementary Material online). Although power to detect a significant departure from the null expectation is low given the small number of coding variants per strain, these findings provide no reason to suspect that the strength of selection against deleterious mutations differs markedly among strains.

**Table 1.** Frequencies of Coding Variants as a Function of Variant Frequency.

	Common Variants^a^ (%; 95% bootstrap CI^b^)	Strain Private Variants (%; 95% bootstrap CI^b^)
Coding variants	75,509 (NA)	439 (NA)
Synonymous variants	49,964 (66.2; 65.8–66.5)	129 (29.4; 25.1–33.7)
Missense variants	25,303 (33.5; 33.1–33.8)	295 (67.2; 62.4–71.4)
Nonsense variants	242 (0.32; 0.28–0.36)	15 (3.4; 1.9–5.3)

a Common variants are defined as variants present in two or more laboratory strains, including the C57BL/6J reference.

b 95% confidence intervals are derived from 1,000 bootstrap resamples of observed variants.

**Fig. 2.**
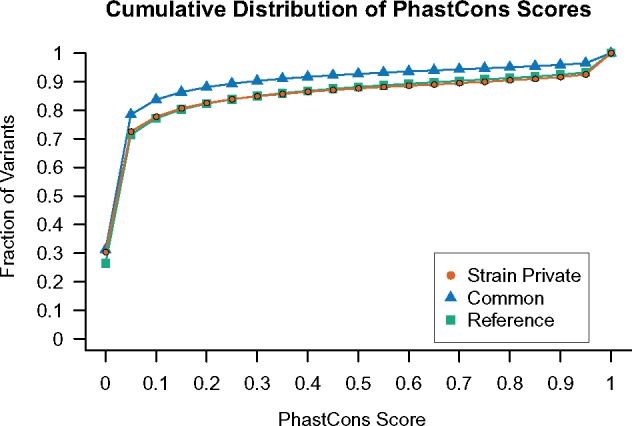
The cumulative distribution of PhastCons conservation scores for strain private and common variants, as well as all sites on the C57BL/6J reference sequence. Common variants are defined as those with the alternative allele present in two or more classical laboratory strains. Only nonrepeat masked sites are considered. The 95% bootstrap confidence intervals associated with each point are narrower than the plotting characters and are therefore omitted from the figure.

Second, I compared the distribution of sequence conservation scores between common and strain-private variants. Sites that are well conserved across species are typically interpreted as targets of purifying selection to maintain a critical biological function (Siepel et al. 2005). Mutations at these sites are expected to be more deleterious, on an average, than those that arise in poorly conserved (and presumably nonfunctional) regions. As a result, evolutionarily conserved sites should be depleted for older, intermediate frequency variants. On the other hand, recent de novo mutations have not yet been strongly shaped by selection, and their genomic distribution should approximate the genome-wide distribution of sequence conservation scores. Consistent with their hypothesized origins from recent mutations, SPVs are enriched in conserved genomic regions compared with common variants (Kolmogorov–Smirnov test *P *<* *2.2×10^−16^), and closely approximate the cumulative distribution of sequence conservation scores across the mouse reference genome (fig. 2). This overall finding is also preserved on a per-strain basis; for all strains, the distribution of sequence conservation scores at SPVs is skewed toward conserved sites relative to common variants (supplementary fig. S3, Supplementary Material online).

The striking enrichment of SPVs in functional coding regions and conserved sequences relative to common variants is consistent with their recent emergence in the laboratory strains. These findings suggest that few ancestral alleles are masquerading as SPVs in this data set and reveal the absence of pervasive, strong selection against new mutations in laboratory colonies. Taken together, these results indicate that the set of SPVs for a focal inbred laboratory strain approximates the cumulative action of diverse germline mutational processes active in that strain.

### The Spectrum of Strain-Specific Variants in Inbred House Mice

**Fig. 3.**
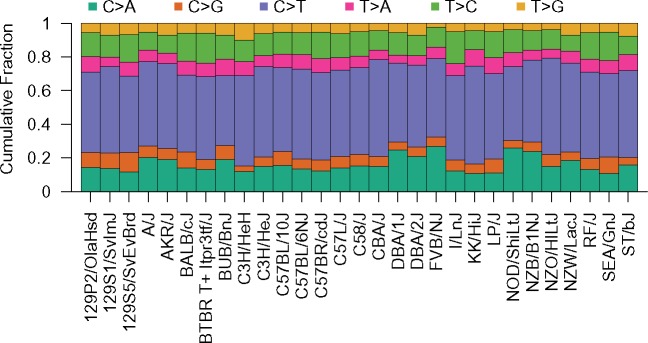
The folded strain private variant spectrum for 29 common inbred laboratory mouse strains. The relative frequency of each mutation type is displayed as a stacked bar plot for each strain.

The most parsimonious interpretation for a SPV on an IBD haplotype is that it arose from a single mutational event during the focal strain’s breeding history. For example, if all classical laboratory strains have a “G” allele at a particular site, with the exception of BALB/cJ which carries a “T” allele, it can be inferred that a G > T mutation occurred in a recent common ancestor of the BALB/cJ strain. By extending this logic genome-wide, I identified the set of single nucleotide mutations that putatively arose in each classical laboratory strain. Using the information available from other strains, I polarized each mutational event into likely ancestral and derived alleles and then quantified the number of mutations in each strain that are of each possible mutational class. To account for the sequence dependency of mutation rates, mutation counts were standardized by the nucleotide composition of all IBD regions in the focal strain and scaled to sum to one. Owing to ambiguity in the strand of origin of a particular mutation, complementary mutations were binned to produce the folded SPV spectrum for each strain (fig. 3).

SPV spectra display qualitative similarities among mouse strains, with relative variant frequencies following the same rank order. For all strains, C > G, T > A, and T > G mutations are the rarest mutational classes, with each mutation type accounting for 2.1–11.7% of SPVs. C > T transitions are the most frequent type of SPVs, ranging from 41.8% to 58.6% of SPVs among strains (fig. 3 and supplementary table S3, Supplementary Material online). This C > T fraction is notably higher than the proportion of rare human SNPs (Harris and Pritchard 2017) and de novo mutations (Rahbari et al. 2016; Jonsson et al. 2017) that are C > T transitions (∼40%), but is consistent with the elevated relative frequency of de novo C > T mutations observed in mouse pedigrees (Lindsay et al. 2018). Despite an overall excess of C > T SPVs, the laboratory mouse strains actually exhibit a marked deficit of CpG>TpG SPVs compared with both de novo mouse and human mutation spectra (supplementary fig. S4, Supplementary Material online). This discrepancy is likely attributable to differences in variant ascertainment between studies, underscoring the need for caution in comparisons of spectra derived from SPVs and de novo mutations. Despite this caveat, mouse SPVs recapitulate key differences between the de novo mutation spectra of mouse and human, including reduced T > C and increased T > A relative mutation frequencies in mouse (supplementary fig. S4, Supplementary Material online; Lindsay et al. 2018).

**Fig. 4.**
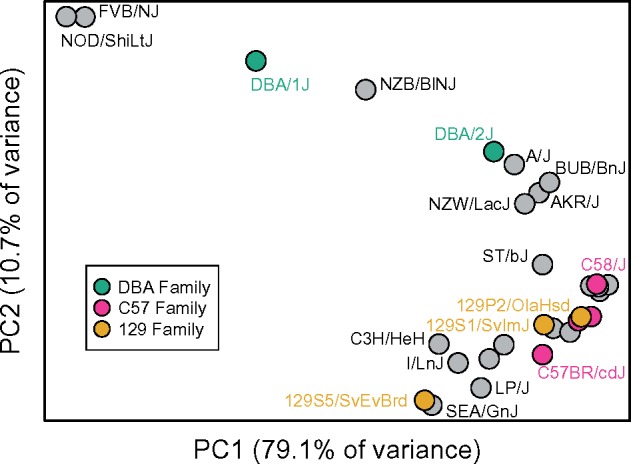
Principle component analysis of strain variation in the SPV spectrum. Closely related strains in the 129, C57, and DBA families are color-coded.

Although the ranked relative frequencies of different variant classes exhibit broad conservation among strains, 71.7% of the 406 possible strain pairs possess significantly distinct SPV spectra (*G*-test, uncorrected *P *<* *0.05, d.f. = 5; supplementary table S6, Supplementary Material online). To further explore this variation, I performed a principle component analysis on the nucleotide-adjusted frequencies of each mutational type across the 29 classical inbred strains. Principle component (PC) 1 isolates FVB/NJ, NOD/ShiLtJ, DBA/1J, and NZB/BlNJ from all other strains (fig. 4). These strains are characterized by an elevated relative rate of C > A mutations and exceptionally low proportions of C > G and T > G mutations (supplementary table S3, Supplementary Material online). Thus, the major axis of variance in these data is dominated by multidimensional properties of the mutation spectrum. Strains belonging to the C57 (C57BL/10J, C57BL/6NJ, C57BR/cdJ, C57L/J) and 129 (129P2/OlaHsd, 129S1/SvImJ, 129S5/SvEvBrd) strain families show a loose tendency to cluster (fig. 4), suggesting that more closely related strains have more similar mutation spectra.

### Causes of Variation in the Mutation Spectrum among Inbred Mouse Strains

The observed strain differences in the mouse mutation spectrum could be driven by environmental differences, strain variation in parental age at reproduction, or genetic factors segregating among strains. Given that inbred strains are reared under standardized laboratory conditions, environmental contributions to strain variation in the mutation spectrum seem unlikely, although effects from minor differences in animal husbandry cannot be ruled out.

There are established age-related shifts in the human de novo mutation spectrum (Jonsson et al. 2017). Although a comparable effect on the mouse mutation spectrum has yet to be shown, differences in reproductive aging between strains could contribute to variation in the mutation spectrum. The inbred strains profiled here significantly differ for two proxy measures of age at reproduction: dam age at first litter (supplementary fig. S5, Supplementary Material online; one-way ANOVA *F*_15,__746_ = 15.11; *P *<* *2.2×10^−16^) and average interbirth interval (supplementary fig. S6, Supplementary Material online; one-way ANOVA *F*_15,2281_= 16.29; *P *<* *2.2×10^−16^). However, variation in these life history traits does not fully account for observed differences in the mutation spectrum. PC1, which captures 79% of the variance in the mutation spectrum among inbred laboratory strains (fig. 4), is not correlated with the dam age at first litter or average interbirth interval (table 2). Similarly, with only one exception, strain variation in the proportion of SPVs belonging to each mutational category is not correlated with either life history trait (table 2). The exception is a positive correlation between the frequency of C > G mutations and interbirth interval (table 2). Many C > G mutations are hypothesized to arise from spontaneous double-strand break induced damage in the germline, the frequency of which may increase with longer generation times (Jonsson et al. 2017; Gao Z, Moorjani P, Amster G, Przeworski M, unpublished data; Agarwal I, Przeworski M, unpublished data).

Taken together, these considerations suggest that the spectrum of SPVs in a given strain is at least partially attributable to segregating genetic differences among strains. Strain variation in the fraction of SPVs within each mutational class is continuous (supplementary fig. S7, Supplementary Material online), suggesting that the genetic control of the germline mutation spectrum is potentially both polygenic and complex.

**Table 2.** Spearman Correlations between Strain Reproductive Traits and the Mutation Spectrum.

	Dam Age at First Litter		Interbirth Interval	
	Spearman’s Rho	*P* value	Spearman’s Rho	*P* value
Principle component 1	0.059	0.831	0.035	0.900
% C>A	−0.026	0.926	−0.421	0.106
% C>G	0.126	0.641	0.647	0.008
% nonCpG>T	0.018	0.952	−0.244	0.361
%CpG>TpG	−0.009	0.978	0.047	0.865
% T>A	0.279	0.294	0.303	0.253
% T>C	0.153	0.571	0.362	0.169
% T>G	−0.203	0.450	0.050	0.857

### Modifiers of the Mouse Mutation Spectrum

My findings raise the possibility that distinct inbred mouse strains harbor unique suites of mutation modifying loci that collectively exert precise, nucleotide-dependent effects on the spectrum of accumulated de novo mutations. Determining the molecular identity of these mutation spectrum modifiers is an important outstanding research aim, albeit one that falls outside the scope of this paper. Genetic differences in genes involved in DNA repair, replication, genome surveillance, and the metabolism of genotoxic compounds pose strong a priori candidates, particularly given their established effects on mutational signatures extracted from human cancers (Nik-Zainal et al. 2012; Alexandrov et al. 2013). Among the 29 mouse strains examined here, there are 845 segregating SNPs that alter the amino acid sequence of genes with GO terms associated with the maintenance of genome integrity, including 8 SNPs with predicted strongly deleterious effects (supplementary table S7, Supplementary Material online). These latter variants present compelling targets for future investigations.

Although the genetic drivers of mutation spectrum heterogeneity remain unknown, many of the causal variants likely derive from the small, ancestral population of (mostly) *M. m. domesticus* mice that provided the genetic source pool for the laboratory inbred strains (Yang et al. 2007). Evolutionary theory predicts considerable scope for segregating mutation modifiers in natural populations (Lynch 2008, 2010; Sung et al. 2012). Although reduced organismal mutation rates are selectively favored in most scenarios (due to the negative fitness consequences of accumulated deleterious alleles), selection against weak mutation rate modifiers is ineffective in small and modestly sized populations, where the stochastic effects of genetic drift overwhelm the deterministic forces of natural selection (Lynch 2010, 2011; Sung et al. 2012). As a result, even moderate-strength modifiers of the mutation spectrum are potentially long-lived in natural mammalian populations and may rise to intermediate allele frequencies. Consistent with this prediction, a recent analysis of haplotype variation in humans reported the action of multiple historical mutation rate modifiers (Seoighe and Scally 2017). These considerations, combined with the large mutational target size for the accumulation of genetic variance for mutation, suggest the high likelihood of ancestrally derived mutation modifiers segregating in inbred mouse strains. Given that laboratory mice capture a limited subset of wild mouse diversity, the magnitude of mutation rate variation in wild populations is almost certainly far greater than that summarized here.

At the same time, mutation modifiers may have also emerged de novo in laboratory colonies. Consistent with this possibility, there are 219 nonsynonymous and premature stop variants in putative mutation modifier candidates that are private to single strains (supplementary table S7, Supplementary Material online).

Further, the efficacy of natural selection against even large-effect mutation rate modifiers in laboratory colonies is likely quite weak due to small effective population sizes and laboratory housing conditions that potentially minimize the negative fitness consequences of deleterious alleles. Thus, both ancestral and young alleles are likely to shape observed variation in the mouse mutation spectrum, but further investigation is required to determine their relative contributions.

Despite these theoretical arguments, there remains little direct evidence for segregating modifiers of the de novo mutation rate in mammalian populations (see Seoighe and Scally 2017 for a notable exception). Mutation rate variation among sequenced human trios can be explained almost entirely by variation in parental age (Kong et al. 2012; Goldman et al. 2016; Jonsson et al. 2017). However, the absence of large-effect modifiers of the *mutation rate* in human populations does not preclude the possibility that loci that exert nuanced effects on the *mutation spectrum* are segregating in our species or present in mice (Harris and Pritchard 2017; Seoighe and Scally 2017).

### Conclusions

Here, I harnessed the unique history of laboratory mice in conjunction with high-quality whole-genome sequences to define SPV spectra in 29 common inbred mouse strains. I documented significant strain variation in the relative probability of different mutational classes, including a strong mutation dependency on local nucleotide context. I showed that SPVs match neutral variant expectations and approximate multidimensional properties of spontaneous germline mutations in house mice. These considerations support the interpretation of SPVs as recent de novo germline mutations. I show that strain variation in age at reproduction cannot explain observed strain differences in the mutation spectrum, demonstrating that the constellation of new mutations that accumulate at a given generation is at least partially subject to genetic control in house mice.

The finding that genetic background likely influences the mutation *spectrum* raises the related question of whether segregating variation also contributes to differences in the overall de novo mutation *rate* among inbred mouse strains. If so, it is of considerable interest to define the genetic architecture of this cellular phenotype, including identifying germline mutation rate modifying genes. Toward this goal, it may be possible to harness genomic resources from The Collaborative Cross (Srivastava et al. 2017) and other recombinant inbred mouse populations to estimate the pace of mutation accumulation in different genetic backgrounds and map global mutation rate modifiers. Notably, the discovery of mutation rate modifiers in mice could steer the search for modifiers in human populations, where the confounding effects of variable mutagen exposure and parental age are likely to impede direct mapping efforts.

## Materials and Methods

### SNP Data and Annotation

Publicly available VCF files from the high-quality whole genome sequences of 29 inbred laboratory mouse strains were downloaded from the Sanger Mouse Genomes project FTP site (ftp://ftp-mouse.sanger.ac.uk/current_snps/; last accessed on May 31, 2017). All genomes were sequenced to >12× coverage, with all but three sequenced to >30× coverage (median coverage = 43.76; supplementary table S3, Supplementary Material online). Variants were identified relative to the GRCm38 reference assembly based on the C57BL/6J inbred mouse strain. Variants were subsequently annotated using snpEff (v4.3t; Cingolani et al. 2012) and intersected with the phastCons60wayPlacental, genomicSuperDups, and RepeatMasker tracks obtained from the UCSC Table Browser (Kent et al. 2002).

The VCF file for 27 wild-caught *M. m. domesticus* mice was obtained from a public repository (http://wwwuser.gwdg.de/∼evolbio/evolgen/wildmouse/; last accessed June 2, 2017 Harr et al. 2016). Variants were filtered to include only biallelic SNPs that pass all filters using VCFtools (Danecek et al. 2011).

De novo mutation data sets for cow (Harland C, Charlier C, Karim L, Cambisano N, Deckers M, Mni M, Mullaart E, Coppieters W, Georges M, unpublished data), human (Jonsson et al. 2017), and mouse (Srivastava et al. 2017; Lindsay et al. 2018) were obtained from the supplemental materials of the associated publications.

### Identification of Strain-Private Substitutions

Shared haplotypes were identified for each pair of laboratory strains using GERMLINE (v1.5.1; Gusev et al. 2008). Briefly, this program identifies genomic regions shared identical by descent (IBD) over a specified minimum block size with a user-defined tolerance for mismatches. Autosomal biallelic SNPs with a minor allele frequency >0.05 across the 29 inbred laboratory strains were used for the inference of IBD haplotypes. A minimum block size of 200 kb and a cutoff of 0 mismatches were specified.

To relate the IBD block sizes in laboratory strains to those found in wild populations, I identified IBD regions in a set of 27 wild-caught *M. m. domesticus* mice from four populations in the native species range (Harr et al. 2016). The largest IBD track found in wild *M. m. domesticus* populations was 4.78 Mb in length. To ensure a focus on IBD regions in the laboratory strains that are shared by virtue of their descent from a single founder animal, I restrict all analyses of mutations in the laboratory strains to IBD regions spanning ≥5 Mb.

I then imposed a set of stringent filters to identify high-quality, SPVs that reside on IBD haplotypes ≥5 Mb:

1. The variant is present as either a heterozygous or fixed singleton in 29 inbred laboratory strain genomes sequenced by the Sanger Mouse Genomes Project
2. The variant passes all filters predefined in the Sanger Mouse Genomes Project VCF file, with the exception of the “Het” filter
3. Variant is a biallelic SNP
4. QUAL >50
5. GQ >60
6. DP >10 and DP <1.9× average sample coverage
7. <15% missing data
8. Site does not overlap segmental duplications annotated in mm10
9. Site is not repeat-masked
10. Genotype likelihood difference >20 between the most likely and next most likely genotype calls in the strain harboring the putative SPV
11. Site is not polymorphic in wild *Mus musculus* or *Mus spretus* genomes (Harr et al. 2016) or present in wild-derived inbred strain genomes (WSB/EiJ, LEWES/EiJ, ZALENDE/EiJ, CAST/EiJ, MOLF/EiJ, PWK/PhJ, and SPRET/EiJ). This filter is imposed to eliminate ancestral variants, but will also remove sites of frequent recurrent mutation (e.g., CpG dinucleotides).
12. If the variant is heterozygous, allele balance ratio >0.3.
13. If the variant is heterozygous, a χ^2^ test on the null hypothesis of no allele bias and no strand bias yields *P* > 0.05

SPVs within IBD regions that passed these filters were then polarized into ancestral and derived states under the assumption that the major (i.e., nonprivate) allele is ancestral. The nucleotides flanking either side of each strain-private variant were extracted from the mm10 reference assembly and used to partition sites into their trinucleotide contexts.

### Identification of Common Mouse Variants

Common variants were defined as biallelic SNPs in the Sanger Mouse Genomes data that are segregating in at least two laboratory strains. Variants in repetitive regions and annotated segmental duplications were excluded to match the filtering criteria employed for the detection of SPVs. The likely ancestral state at each site was defined using parsimony. Briefly, I identified the subset of common laboratory strain variants that are fixed for a single allele in the wild-derived inbred strains PWK/PhJ (*M. m. musculus*), CAST/EiJ (*M. m. castaneus*), and SPRET/EiJ (*M. spretus*). In these cases, the ancestral state is inferred to be the allele present in the wild-derived samples.

### Strain Breeding Characteristics

Measures of strain breeding performance were obtained from the JAX5 database (Currer et al. 2009) downloaded from the Mouse Phenome Database (Bogue et al. 2018). One-way ANOVA tests treating strain as a factor were used to test for significant among-strain variation in measures related to reproductive aging.

### Statistical Analyses

All analyses were carried out in the R environment for statistical computing (R Core Team 2016). Mutational spectra were compared using *G*-tests of independence. Principle component analysis was carried out on untransformed relative frequencies of six mutational classes (standardized by the nucleotide content of IBD regions) using the prcomp function in R. Data were zero-centered and scaled to have unit variance prior to analysis.

## Supplementary Material

Supplementary Data
