# Supplementary material for: Significant Strain Variation in the Mutation Spectra of Inbred Laboratory Mice
Source: Mol Biol Evol. 2019 Feb 11;36(5):865–74. doi: 10.1093/molbev/msz026 (PMC6501876; doi:10.1093/molbev/msz026)

SUPPLEMENTARY MATERIALS

Figure S1. The distribution of pairwise IBD (autozygous) block sizes in 29 inbred strains and 27 wild-caught *M. m. domesticus* mice. No blocks larger than 5Mb are observed in wild samples, but the distribution of IBD blocks in the inbred strains was clipped to <10Mb for visualization.

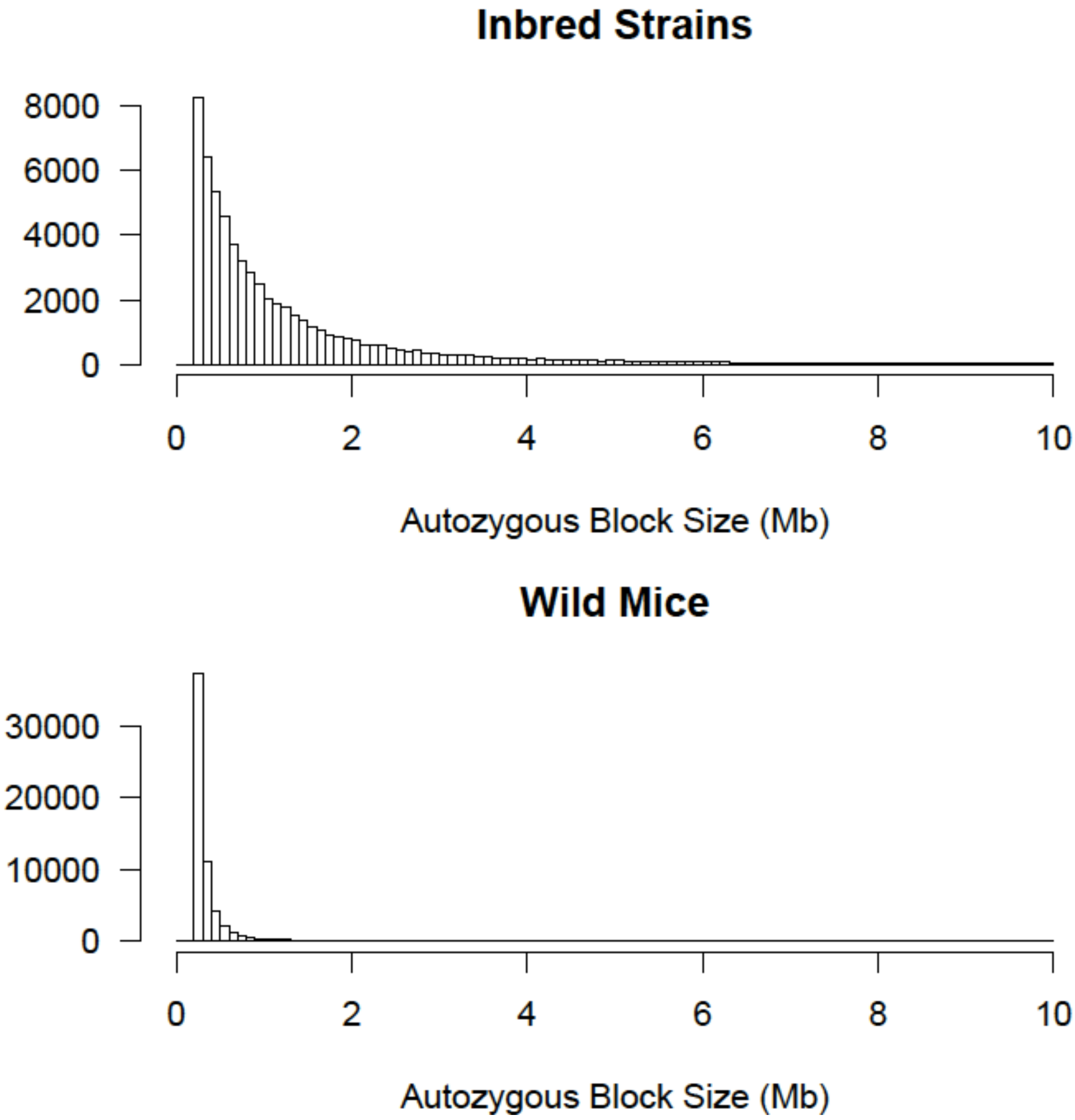

Figure S2. Stacked bar graphs depicting the fraction of strain private coding variants that are synonymous, missense, or nonsense for inbred mouse strains. The corresponding fractions of coding mutations are also given for common mouse variants segregating in at least 2 inbred strains. The expected frequencies of each type of coding mutation under neutral evolution is also given.

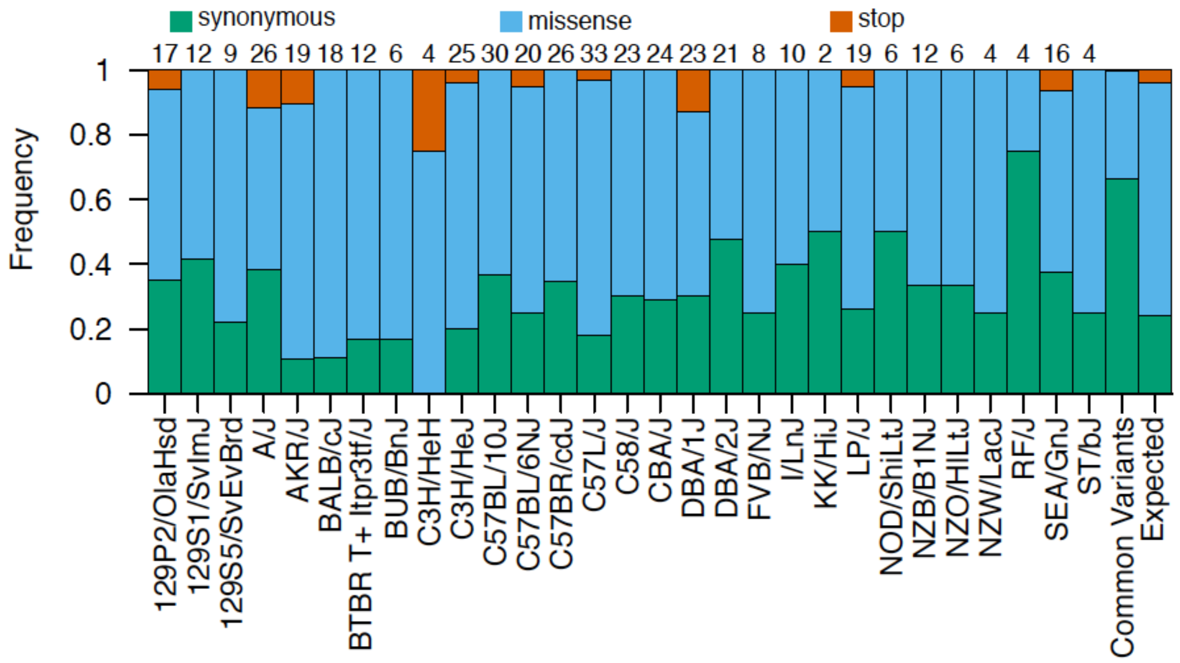

Figure S3. The cumulative distribution of PhastCons conservation scores for the set of strain private variants ascertained in each of the 29 inbred mouse strains. Cumulative distributions are also provided for common variants (circles) and non-repeat masked sites across the mm10 reference genome assembly (squares).

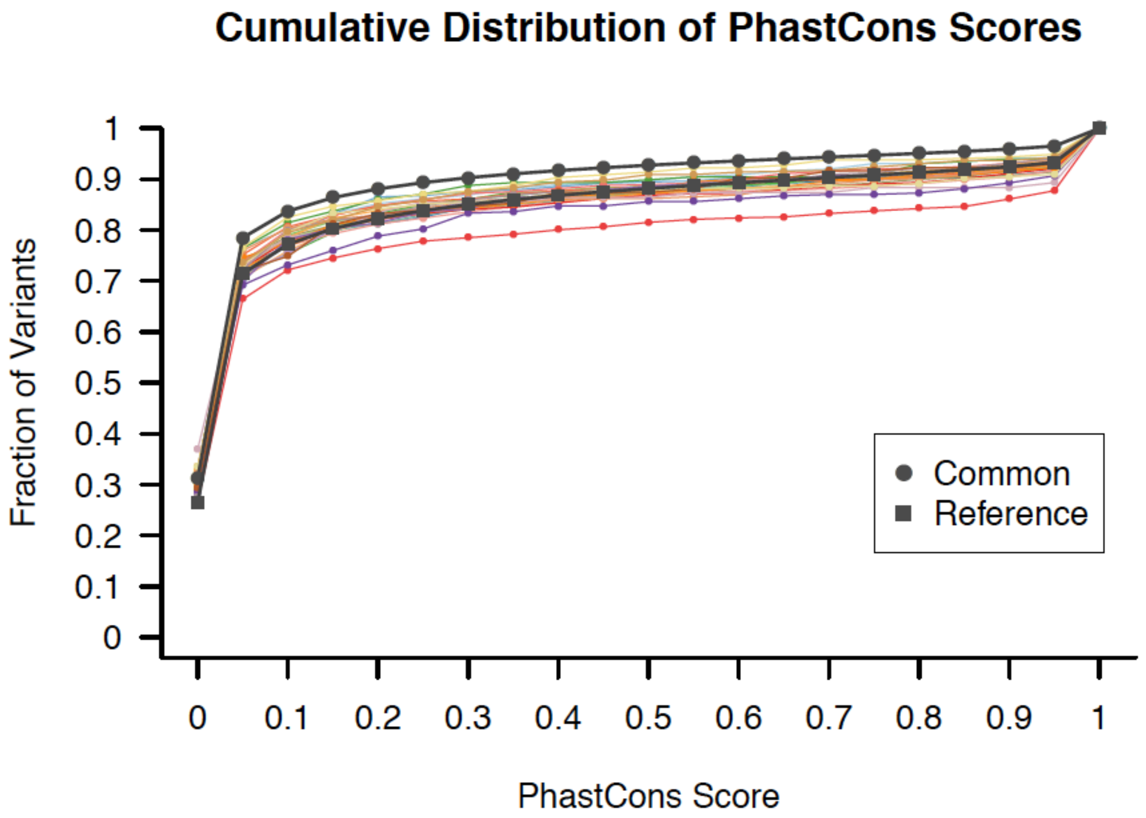

Figure S4. Comparison of the laboratory mouse strain private substitution mutation spectrum with human, cow, and mouse *de novo* SNP calls. Confidence intervals were derived by 1000 bootstrap resamples of empirical SNP calls followed by re-estimation of the mutation spectrum.

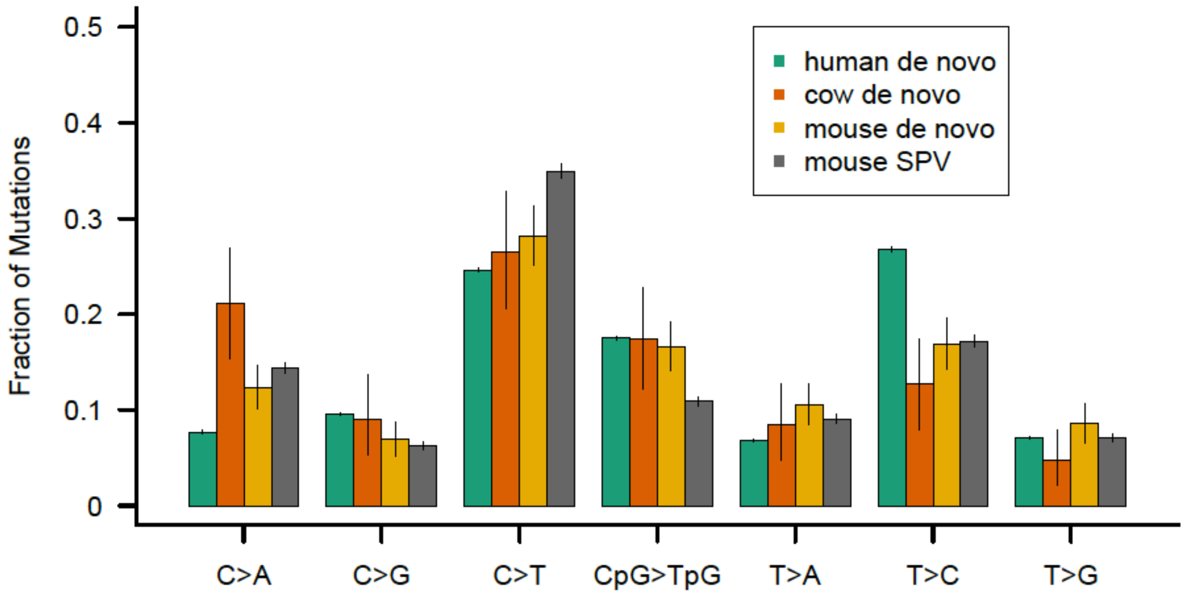

Figure S5. Mean dam age at first litter (+/- 1 standard deviation) for inbred mouse strains. Data not available for all strains.

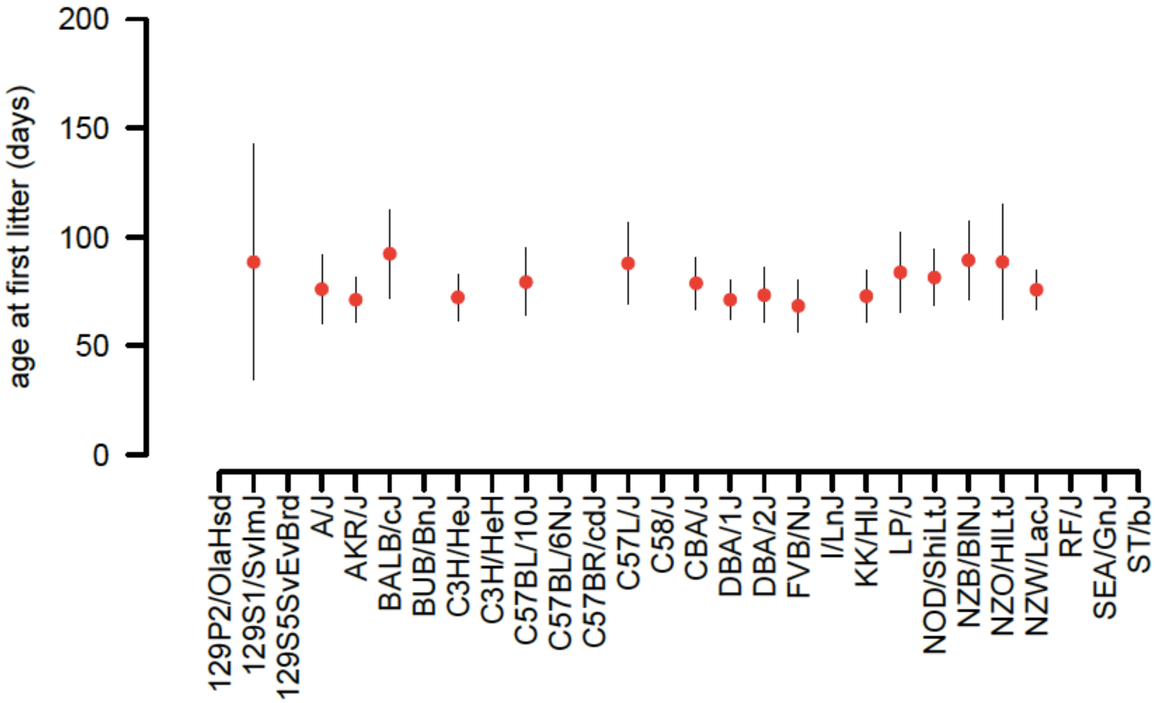

Figure S6. Mean interbirth interval (+/- 1 standard deviation) for inbred mouse strains. Data not available for all strains.

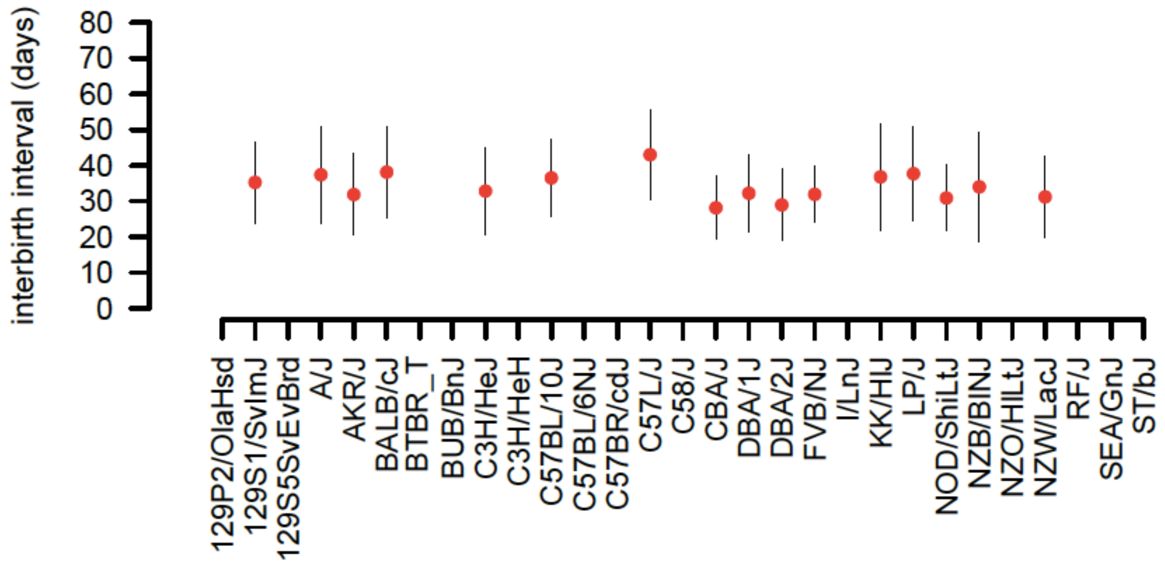

Figure S7. Relative frequencies of each mutational class across the 29 inbred mouse strains. Error bars correspond to 95% bootstrap confidence intervals derived from 1000 bootstrap permuations of the observed data.

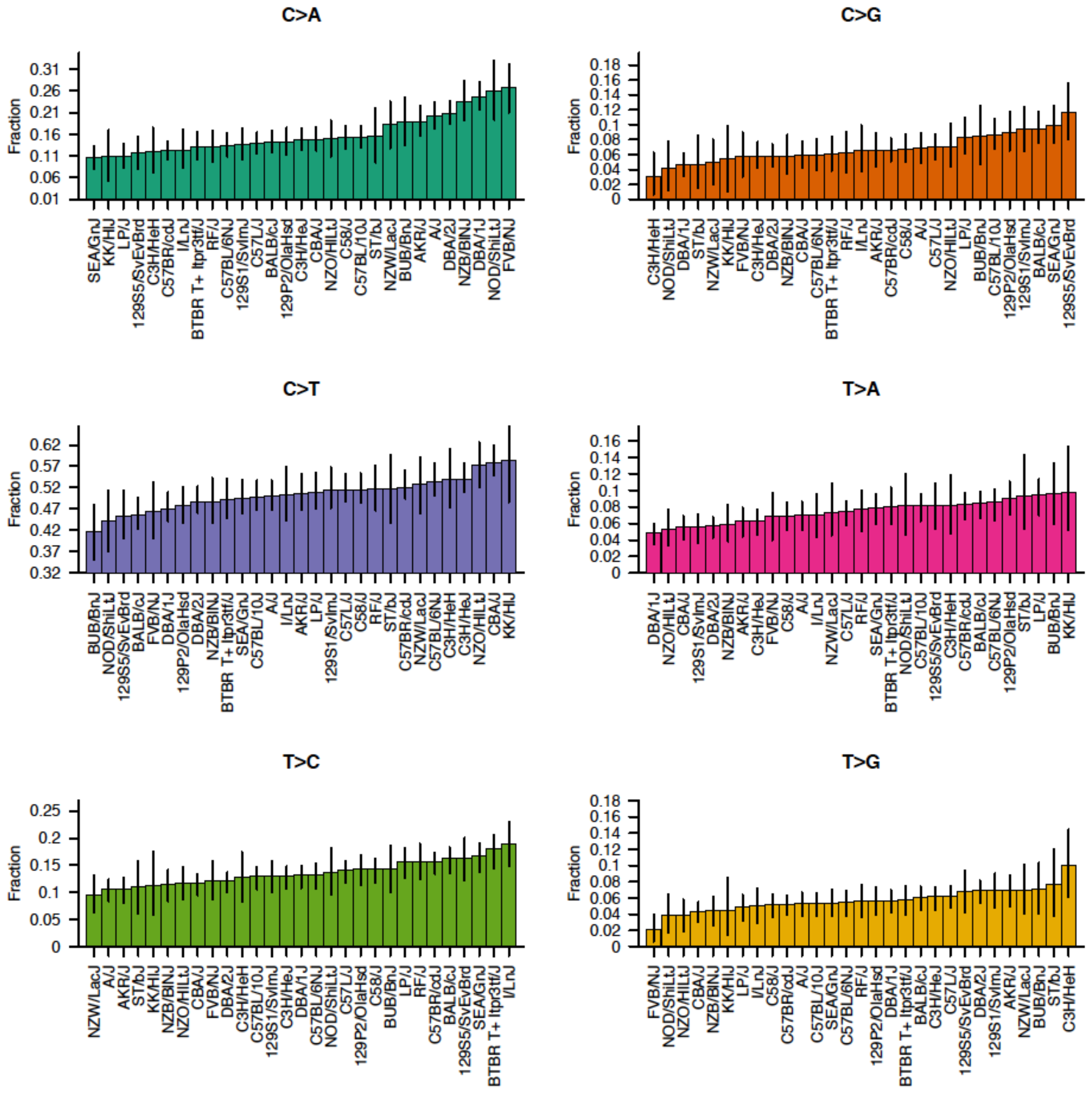

Supplement: Supplementary Data [file msz026_supp.zip › supplementary_materials.pdf]
